# Identifying predictors of student depression through validated machine learning pipelines

**DOI:** 10.3389/fmed.2026.1864665

**Published:** 2026-06-29

**Authors:** Jacob Washton, Tracy Owens, Antony Sierra, Milan Toma

**Affiliations:** 1 Algorithmic Medicine Laboratory, Department of Osteopathic Manipulative Medicine, College of Osteopathic Medicine, New York Institute of Technology, Old Westbury, NY, United States; 2 Office of Academic Enrichment, College of Osteopathic Medicine, New York Institute of Technology, Jonesboro, AR, United States; 3 NYS-OMH Pilgrim Psychiatric Center, Brentwood, NY, United States

**Keywords:** feature importance, hyperparameter optimization, learning curves, machine learning, mental health prediction, model validation, RUSBoost, student depression

## Abstract

**Background:**

Depression among student populations has become a growing public health concern, with prevalence rates ranging from 10 to 30 percent across studies. Machine learning methods offer the potential not only to predict depression risk but also to identify which factors most strongly predict depression through feature importance analysis. However, the validity of such rankings depends critically on the quality of the underlying model's learning dynamics; which is a consideration often overlooked when aggregate performance metrics appear favorable.

**Methods:**

This study evaluated six baseline classification algorithms (Logistic Regression, Decision Tree, k-Nearest Neighbors, Support Vector Machine, Naive Bayes, and Random Forest) and two RUSBoost pipeline configurations on a student depression dataset comprising 27,901 records. Pipeline A employed fixed hyperparameters with constant model complexity during learning curve construction, while Pipeline B implemented systematic hyperparameter optimization through grid search and scaled model complexity proportionally with training data availability. Learning curves were generated by training models on progressively larger subsets of training data (10% to 100%) to assess whether each algorithm exhibited healthy learning dynamics characterized by monotonically increasing validation accuracy and convergent training-validation gaps.

**Results:**

Despite achieving the highest F1-score (87.5%), Logistic Regression and other baseline algorithms exhibited pathological learning dynamics including flat curves indicating no benefit from additional training data, severe overfitting with training-validation gaps exceeding 15 percentage points, and erratic non-monotonic behavior. Pipeline A's RUSBoost implementation showed oscillatory validation accuracy and failed to converge to a stable asymptote. Only Pipeline B demonstrated textbook healthy learning dynamics: training accuracy decreased monotonically from 88.8% to 85.4% while validation accuracy increased monotonically from 82.2% to 83.1%, with progressive gap convergence. Feature importance analysis from the validated Pipeline B model identified history of suicidal thoughts as the dominant predictor (normalized importance: 1.0), followed by academic pressure (0.57), financial stress (0.31), age (0.18), work/study hours (0.13), dietary habits (0.11), and study satisfaction (0.07).

**Conclusions:**

This study demonstrates that aggregate performance metrics are insufficient indicators of model reliability for scientific inference. Learning curve diagnostics must precede interpretation of feature importance rankings to ensure conclusions rest on demonstrably healthy learning processes rather than artifacts of pathological training dynamics. The validated model's identification of suicidal ideation history, academic pressure, and financial stress as leading predictors suggests that targeted screening for suicidal thoughts, academic workload management programs, and financial support initiatives may prove most effective for reducing depression prevalence among student populations.

## Graphical Abstract

**Figure:**
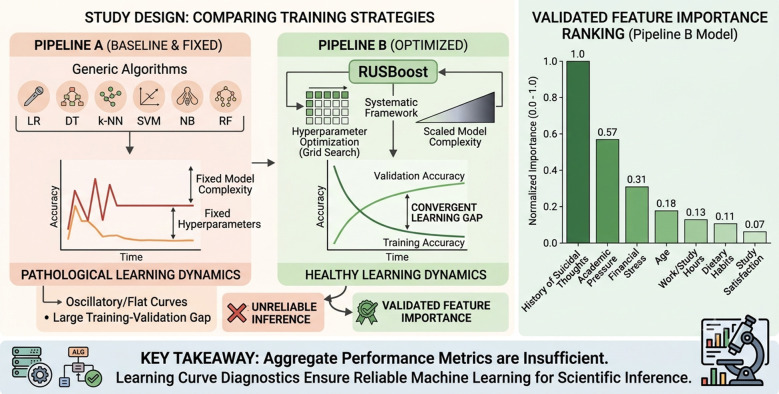

## 1 Introduction

Depression is a psychiatric disorder characterized by persistent feelings of sadness, hopelessness, and loss of interest in activities (1). The condition has been recognized since antiquity, with descriptions appearing in ancient Greek medical texts where it was termed melancholia and attributed to an imbalance of bodily humors (2, 3). Modern clinical understanding of depression emerged in the nineteenth and twentieth centuries as the disorder was distinguished from other mood disturbances and codified in diagnostic classification systems (4). Today, depression is recognized as one of the leading causes of disability worldwide, affecting an estimated 280 million people globally according to the World Health Organization (5).

The prevalence of depression among student populations has become a matter of increasing concern. University and college students face a unique combination of stressors including academic pressure, financial strain, social adjustment, and uncertainty about future employment (6, 7). Studies conducted across multiple countries have reported depression prevalence rates among students ranging from 10 to 30 percent, with some surveys indicating even higher rates during examination periods or following major life transitions (8, 9). The consequences of untreated depression in this population extend beyond immediate suffering to include impaired academic performance, increased dropout rates, substance abuse, and in severe cases, suicidal ideation and behavior (10). Early identification of students at risk for depression is therefore a priority for educational institutions and public health systems (11–13).

Medical students specifically face distinct challenges beyond typical student stressors. As former high-achievers accustomed to academic excellence, they encounter a qualitative shift in medical school, e.g., accelerated pace, interleaved topics, and cognitive demands that exceed prior memorization strategies (14). When placed among equally prepared peers, students who were consistently top performers may struggle for the first time, triggering self-doubt and imposter syndrome (15, 16). Cultural and familial expectations, particularly from physician families, compound these pressures, creating fear of disappointing relatives or violating cultural norms (17, 18). Financial considerations intensify these challenges. With medical education debt reaching hundreds of thousands of dollars, students face a psychological bind: withdrawing leaves them with enormous debt and no credential, creating feelings of entrapment and desperation (19, 20). Because these students have historically been academically successful, seeking help feels like admitting weakness, leading many to suffer in isolation (21, 22). The relationship between academic performance and mental health is bidirectional and self-reinforcing: poor performance precipitates depression, while depression impairs cognitive function and academic performance (23, 24). Early identification through machine learning models offers systematic detection that does not require students to self-disclose their struggles, potentially interrupting this destructive cycle.

Machine learning methods have been applied to mental health research with increasing frequency over the past two decades (25). Early applications focused on text analysis, using natural language processing to detect depressive symptoms in clinical notes, social media posts, and online forum discussions (26). Subsequent work expanded to include structured data from electronic health records, wearable devices, and survey instruments. Classification algorithms including logistic regression, support vector machines, random forests, and neural networks have been employed to predict depression diagnoses, treatment response, and relapse risk (27–29). Several studies have demonstrated that machine learning models can achieve diagnostic accuracy comparable to or exceeding that of traditional screening instruments when trained on sufficiently large and representative datasets (30, 31).

However, the potential contribution of machine learning to depression research extends beyond the development of screening tools. A well trained classification model does more than assign risk categories to individuals. The model also learns which input features are most predictive of the outcome, effectively ranking the variables in the dataset by their importance for distinguishing between depressed and non-depressed individuals. In the context of student depression, this capability is particularly valuable. A feature importance analysis can reveal whether academic factors such as grade point average and study satisfaction are more or less predictive than lifestyle factors such as sleep duration and dietary habits, or whether demographic and family history variables dominate the prediction. An additional complexity in depression research concerns psychosomatic manifestations, where depression and stress exhibit as physical symptoms. Students experiencing mental health challenges may report physical complaints (e.g., chronic fatigue, digestive issues, headaches) while the underlying etiology is psychological (32, 33). Machine learning algorithms trained on expanded datasets that include both psychological and physical symptom features could potentially identify such latent relationships, revealing patterns where clusters of physical symptoms serve as proxies for undiagnosed mental health conditions (34, 35). This capability would be particularly valuable for early detection, as students may seek medical attention for physical symptoms before recognizing or acknowledging psychological distress. Such rankings provide clinical insight into the determinants of depression within the student population and can inform the design of targeted intervention programs. If academic pressure emerges as a dominant predictor, institutions might prioritize workload management and examination reform. If sleep duration ranks highly, public health campaigns promoting healthy sleep habits may be warranted. The feature importance output of a machine learning model thus serves as an empirical window into the etiology of depression among students.

The validity of feature importance rankings depends critically on the quality of the underlying model (36). A classifier that has been poorly trained, overfit to idiosyncratic patterns in the training data, or optimized with inappropriate hyperparameters cannot be trusted to produce meaningful importance estimates. The rankings derived from such a model would reflect artifacts of the training process rather than genuine relationships between predictors and outcome. For this reason, rigorous validation of learning dynamics is a prerequisite for interpreting feature importance results. Only when the training process exhibits healthy characteristics, including monotonically improving validation accuracy, convergent training and validation curves, and appropriate scaling of model complexity with data availability, can researchers confidently draw substantive conclusions from the model's internal structure.

A model may achieve high accuracy while exhibiting pathological (i.e., non-convergent, erratic, or unstable) learning dynamics (36). Such dynamics include training accuracy that remains constant regardless of data volume, validation accuracy that decreases with additional training data, or a persistent large gap between training and validation performance. When these patterns emerge, the resulting model cannot be trusted for deployment or for drawing scientific conclusions about feature importance, irrespective of how favorable the final metrics appear.

This paper compares two machine learning pipeline configurations applied to student depression classification, demonstrating how methodological choices in model training directly impact the interpretability and trustworthiness of learning dynamics and, consequently, the reliability of feature importance analyses.

## 2 Material and methods

### 2.1 Dataset

This study utilizes the Student Depression Dataset, a publicly available resource designed for analyzing mental health trends and predictors among students (37). The dataset compiles comprehensive information to support research in psychology, data science, and education, with the goal of understanding factors that contribute to student mental health challenges and informing early intervention strategies.

The dataset is provided in CSV format, where each row represents an individual student record. Table 1 summarizes the features included in the dataset.

**Table 1:** Feature descriptions for the Student Depression Dataset.

Feature	Category	Description
ID	Identifier	Unique identifier assigned to each student record
Gender	Demographic	Gender of the student (Male, Female, Other)
Age	Demographic	Age of the student in years
City	Demographic	City or region of residence
Profession	Academic	Field of work or study
Degree	Academic	Academic degree or program pursued
CGPA	Academic	Cumulative grade point average
Academic Pressure	Academic	Level of pressure from academic expectations
Study Satisfaction	Academic	Satisfaction level with studies
Work Pressure	Lifestyle	Pressure related to job responsibilities
Job Satisfaction	Lifestyle	Satisfaction with work environment
Work/Study Hours	Lifestyle	Average daily hours dedicated to work or study
Sleep Duration	Lifestyle	Average hours of sleep per day
Dietary Habits	Lifestyle	Assessment of eating patterns and nutrition
Financial Stress	Wellbeing	Stress experienced due to financial concerns
Family History of Mental Illness	Wellbeing	Indicates family history of mental illness (Yes/No)
Suicidal Thoughts History	Wellbeing	Whether the student has experienced suicidal ideation (Yes/No)
Depression	Target	Binary indicator of depression status (Yes/No)

The target variable, *Depression*, is a binary indicator denoting whether a student is experiencing depression. This variable serves as the primary outcome for the classification task. The dataset integrates demographic, academic, and lifestyle factors to provide a multifaceted view of student wellbeing, enabling analysis of how variables such as academic pressure, sleep duration, financial stress, and family history interact to influence mental health outcomes (37).

The Student Depression Dataset is a synthetic dataset created for machine learning research and educational purposes rather than a clinical dataset derived from human subjects research. As such, conventional provenance information applicable to clinical datasets (including geographic origin, institutional source, recruitment strategy, inclusion/exclusion criteria, ethics committee approval, and participant consent procedures) does not apply. The binary depression indicator was generated algorithmically based on weighted combinations of the predictor variables, designed to simulate realistic feature-outcome relationships for machine learning benchmarking purposes; it does not represent clinical diagnoses derived from validated instruments or clinician assessment. Because no human subjects were involved in data generation, institutional review board approval was neither required nor applicable for either the original dataset creation or the present secondary analysis. Importantly, because the dataset is synthetically generated, it has no geographic provenance, i.e., the data do not originate from any real-world location, population, or data collection effort. The synthetic nature of the dataset does not compromise the methodological objectives of this study, which focused on demonstrating the importance of validated learning dynamics for trustworthy feature importance analysis. However, researchers should recognize that findings derived from synthetic data require validation on real-world clinical datasets from diverse populations before informing actual screening or intervention practices.

Beyond classification accuracy, a key output of well-trained machine learning models is the feature importance ranking, which reveals how the algorithm prioritizes different predictors when making decisions. For mental health research, this ranking is particularly informative: it can illuminate which factors (e.g., academic pressure, sleep duration, financial stress, family history, or others) most strongly predict depression among students. However, the validity of these importance rankings depends entirely on the integrity of the training process. A model exhibiting pathological learning dynamics, such as flat training curves, erratic validation performance, or persistent overfitting, cannot be trusted to produce meaningful feature rankings regardless of its apparent predictive accuracy (38). This is precisely why rigorous attention to learning curve diagnostics is vital: only when we can demonstrate healthy learning behavior (i.e., monotonically improving validation accuracy, convergent training-validation gaps, and appropriate scaling of model complexity with data availability) can we confidently interpret the resulting feature importance plot as a reliable guide to the factors underlying student depression (39). The methodological rigor applied to validating training dynamics is thus not merely a technical exercise but a prerequisite for drawing substantive scientific conclusions from the model's internal structure.

#### 2.1.1 Class imbalance assessment

Preliminary analysis of the Student Depression Dataset revealed class imbalance in the target variable. Of the 27,901 student records, 11,565 (41.5%) were labeled as non-depressed and 16,336 (58.5%) as depressed, yielding an imbalance ratio of approximately 1.41:1. While this ratio is moderate compared to severely imbalanced datasets encountered in other medical domains, it is sufficient to bias standard classification algorithms toward the majority class, potentially compromising recall for non-depressed students and distorting feature importance rankings.

### 2.2 Baseline algorithm evaluation

Prior to selecting a final classification algorithm, six candidate techniques were evaluated: Logistic Regression, Decision Tree, k-Nearest Neighbors, Support Vector Machine, Naive Bayes, and Random Forest. These algorithms span different learning paradigms including linear methods, instance-based learning, probabilistic classifiers, kernel methods, and ensemble approaches.

Table 2 summarizes the hyperparameter configurations used for each baseline algorithm. Logistic Regression employed ridge regularization (L2 penalty) with the logistic loss function. Decision Tree was configured with a maximum of 20 splits and a minimum leaf size of 5 instances. k-Nearest Neighbors used 5 neighbors with Euclidean distance and no internal standardization, as features were pre-standardized during preprocessing. Support Vector Machine employed a radial basis function kernel with box constraint parameter set to 1. Naive Bayes used kernel density estimation rather than parametric Gaussian assumptions to accommodate potentially non-normal feature distributions. Random Forest was implemented using bagging with 100 learning cycles and decision tree weak learners. These configurations represent commonly used default or near-default values, enabling a fair baseline comparison against which the RUSBoost pipelines could be evaluated.

**Table 2:** Hyperparameter configurations for baseline algorithms.

Algorithm	Hyperparameters
Logistic Regression	Learner: logistic; regularization: ridge (L2)
Decision Tree	MaxNumSplits: 20; MinLeafSize: 5
k-Nearest Neighbors	NumNeighbors: 5; Distance: Euclidean; Standardize: false
Support Vector Machine	KernelFunction: RBF; BoxConstraint: 1; Standardize: false
Naive Bayes	DistributionNames: kernel (kernel density estimation)
Random Forest	Method: Bag; NumLearningCycles: 100; Learners: tree

The evaluation protocol proceeded in two stages. The first stage involved training each algorithm on the full training set and computing standard performance metrics on the held-out test set, including accuracy, precision, recall, F1-score, and area under the receiver operating characteristic curve. The second stage involved generating learning curves by training models on progressively larger subsets of the training data, ranging from 0.1 to 1.0 in increments of 0.1. At each fraction, stratified sampling preserved the original class distribution, and five repetitions were performed to reduce variance. Both training and validation accuracy were recorded at each training set size.

The learning curves were examined for indicators of healthy and pathological behavior. Healthy dynamics were characterized by monotonically increasing validation accuracy, decreasing gaps between training and validation accuracy, and convergence toward a common asymptote. Pathological patterns included flat or erratic validation curves, decreasing validation accuracy with additional data, and persistent large training-validation gaps. Algorithms exhibiting pathological dynamics were considered unsuitable regardless of aggregate metrics. Only algorithms demonstrating healthy learning dynamics were considered candidates for final selection, with aggregate metrics used as secondary criteria.

### 2.3 RUSBoost as contingency classifier

Should the baseline algorithms exhibit pathological learning dynamics or fail to achieve satisfactory performance, RUSBoost will be adopted as an alternative approach. RUSBoost combines random under-sampling with the AdaBoost boosting framework, creating balanced training subsets at each boosting iteration. The boosting framework iteratively adjusts instance weights to focus learning on difficult-to-classify examples. This combination can produce more stable learning dynamics and feature importance rankings that are robust to training set composition. Given a training set $D={\left\{\left({\text{x}}_{i},y_{i}\right)\right\}}_{i=1}^{N}$ where *y*_*i*_∈{0, 1}, the algorithm iteratively trains weak learners on balanced subsets created through under-sampling of the majority class.

At iteration *t*, the algorithm performs the following operations. First, it applies random under-sampling to the current weighted distribution to obtain a balanced subset $D_{t}^{\prime }$. Second, it trains a weak learner *h*_*t*_ on $D_{t}^{\prime }$. Third, it computes the pseudo-loss (Equation 1):


$$\begin{array}{l} \epsilon_{t}=\underset{i:h_{t}\left({\text{x}}_{i}\right)\neq y_{i}}{\sum }D_{t}\left(i\right) \end{array}$$ (1)

where *D*_*t*_(*i*) represents the weight of instance *i* at iteration *t*. Fourth, it calculates the learner weight (Equation 2):


$$\begin{array}{l} \alpha_{t}=\frac{1}{2}\ln\left(\frac{1-\epsilon_{t}}{\epsilon_{t}}\right) \end{array}$$ (2)

Finally, it updates instance weights according to Equation 3:


$$\begin{array}{l} D_{t+1}\left(i\right)=\frac{D_{t}\left(i\right)\exp\left(-\alpha_{t}y_{i}h_{t}\left({\text{x}}_{i}\right)\right)}{Z_{t}} \end{array}$$ (3)

where *Z*_*t*_ is a normalization constant. The final ensemble prediction is given by Equation 4:


$$\begin{array}{l} H\left(\text{x}\right)=\text{sign}\left(\overset{T}{\underset{t=1}{\sum }}\alpha_{t}h_{t}\left(\text{x}\right)\right) \end{array}$$ (4)

### 2.4 Learning curves and model diagnostics

A learning curve plots model performance against the number of training examples. For a training set of size *n*, denote the training accuracy as Acc_train_(*n*) and validation accuracy as Acc_val_(*n*). Healthy learning dynamics exhibit the following characteristics:

1. Training accuracy starts high for small *n* and may decrease slightly as *n* increases due to reduced overfitting capacity.
2. Validation accuracy starts lower than training accuracy and increases monotonically toward an asymptote.
3. The gap Acc_train_(*n*)−Acc_val_(*n*) decreases as *n* increases, indicating convergence.

These properties can be formalized. As *n* → ∞, both curves should converge Equation 5:


$$\begin{array}{l} \underset{n\to \infty }{\lim}\left[{\text{Acc}}_{\text{train}}\left(n\right)-{\text{Acc}}_{\text{val}}\left(n\right)\right]=0 \end{array}$$ (5)

When this convergence fails to occur, or when the validation curve exhibits non-monotonic behavior, the learning process is considered unhealthy and results cannot be trusted.

### 2.5 Data preprocessing pipeline

Both implementations share an identical preprocessing pipeline. The student depression dataset undergoes the following transformations. Missing numeric values are imputed using the median of each respective feature. Missing categorical values are imputed using the mode. Binary variables including Gender and suicidal thought history are encoded as numeric indicators. Ordinal variables such as Dietary Habits are mapped to integer scales preserving order relationships. High-cardinality categorical features including Profession, City, and Degree are removed to prevent sparse representations. Features identified as having low predictive importance in preliminary analysis are excluded. All remaining features are standardized using z-score normalization (Equation 6):


$$\begin{array}{l} {\tilde{x}}_{j}=\frac{x_{j}-\mu_{j}}{\sigma_{j}} \end{array}$$ (6)

where μ_*j*_ and σ_*j*_ represent the mean and standard deviation of feature *j* computed on the training set.

### 2.6 Data partitioning

The dataset is partitioned into three disjoint subsets using stratified sampling to preserve class proportions. The training set comprises 70% of the original data. The remaining 30% is split equally between validation and test sets, yielding 15% each. This partitioning scheme ensures that hyperparameter selection (using validation data) remains independent of final performance evaluation (using test data).

Let *N* denote the total number of instances. The partition sizes are (Equations 7–9):


$$\begin{array}{ll} N_{\text{train}} & =\left\lfloor 0.70\cdot N\right\rfloor \end{array}$$ (7)

$$\begin{array}{ll} N_{\text{val}} & =\left\lfloor 0.50\cdot \left(N-N_{\text{train}}\right)\right\rfloor \end{array}$$ (8)

$$\begin{array}{ll} N_{\text{test}} & =N-N_{\text{train}}-N_{\text{val}} \end{array}$$ (9)

### 2.7 Class weighting

To further address class imbalance, instance weights are assigned inversely proportional to class frequency (Equation 10):


$$\begin{array}{l} w_{i}=\frac{1}{\left|\left\{j:y_{j}=y_{i}\right\}\right|} \end{array}$$ (10)

This weighting scheme ensures that minority class instances exert greater influence on the learning objective.

### 2.8 Hyperparameter configurations

#### 2.8.1 Pipeline A: fixed

The first implementation employs predetermined hyperparameter values without optimization. The configuration is summarized in Table 3.

**Table 3:** Fixed hyperparameter configuration for Pipeline A.

Hyperparameter	Symbol	Value
Number of Learning Cycles (Main Model)	*T*	150
Number of Learning Cycles (Submodels)	*T*_sub_	120
Learning Rate	η	0.1
Maximum Tree Splits	*S*_max_	20
Under-sampling Ratio	*r*	1 (default)

The learning curve construction in Pipeline A trains submodels with identical complexity regardless of training subset size. For each fraction *f*∈{0.1, 0.2, …, 1.0} of the training data, a RUSBoost model with *T*_sub_ = 120 cycles is trained. This approach treats model complexity as independent of available data.

#### 2.8.2 Pipeline B: optimized

The second implementation performs systematic hyperparameter search over a predefined grid. The search space is defined in Table 4.

**Table 4:** Hyperparameter search space for Pipeline B.

Hyperparameter	Symbol	Candidate values
Number of Learning Cycles	*T*	{100, 200, 400}
Learning Rate	η	{0.05, 0.1}
Maximum Tree Splits	*S*_max_	{10, 20, 40}
Under-sampling Ratio	*r*	{1, 2}

The grid search evaluates |*T*| × |η| × |*S*_max_| × |*r*| = 3 × 2 × 3 × 2 = 36 configurations. For each configuration θ = (*T*, η, *S*_max_, *r*), a model *M*_θ_ is trained on the training set and evaluated on the validation set. The optimal configuration is Equation 11:


$$\begin{array}{c} \theta^{*}=\underset{\theta }{\mathrm{argmax}}{\text{Acc}}_{\text{val}}\left(M_{\theta }\right) \end{array}$$ (11)

A critical distinction in Pipeline B concerns learning curve construction. Rather than using fixed model complexity, the number of boosting cycles scales with the training fraction Equation 12:


$$\begin{array}{c} T_{\text{scaled}}\left(f\right)=\max\left(30,\left\lfloor T^{*}\cdot f\right\rfloor \right) \end{array}$$ (12)

where T^*^ is the optimal number of cycles from grid search and *f* is the fraction of training data used. This scaling ensures that model capacity grows proportionally with available data, preventing the artificial inflation of training accuracy on small subsets.

#### 2.8.3 Sensitivity analysis for minimum epoch threshold

To assess whether the choice of minimum epoch threshold materially affects learning curve characteristics, a sensitivity analysis is conducted. Learning curves are generated using minimum thresholds of 10, 20, 30, 40, and 50 epochs, with all other hyperparameters held constant at their optimized values from grid search. For each threshold value, the complete learning curve construction procedure (training fractions from 0.1 to 1.0, five repetitions per fraction, stratified sampling) is executed identically. Four metrics are computed for each threshold: final validation accuracy at 100% training data, final training-validation gap, mean validation accuracy across all fractions, and the number of monotonicity violations (defined as instances where validation accuracy decreased between consecutive training fractions). The coefficient of variation across thresholds is calculated to quantify overall sensitivity.

### 2.9 Configuration workflows

**Figure 1:**
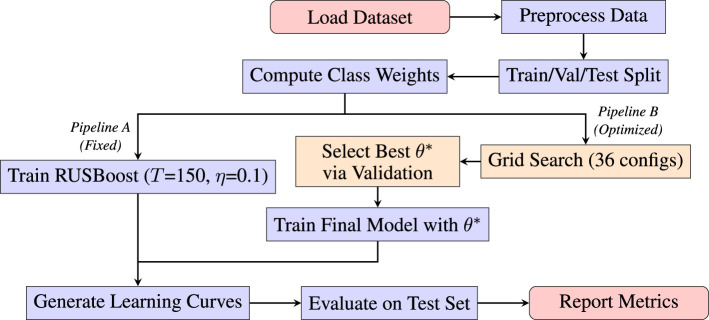
Unified workflow diagram showing shared preprocessing and evaluation stages with divergent training pipelines (**(A)** fixed hyperparameters; **(B)** grid search optimization). Orange blocks indicate the tuning phase in Pipeline B.

Figure 1 illustrates the unified workflow for both pipeline configurations. Both pipelines share identical preprocessing stages: data loading, preprocessing, train/validation/test partitioning (70/15/15), and class weight computation. The workflows diverge at the model training phase. Pipeline A proceeds directly to training a RUSBoost model with fixed hyperparameters (*T* = 150, η = 0.1). Pipeline B first performs a grid search over 36 hyperparameter configurations, selects the optimal configuration θ^*^ based on validation accuracy, and then trains the final model using the selected parameters. Both pipelines converge for learning curve generation and test set evaluation, though their approaches to learning curve construction differ as detailed in the Supplementary material.

### 2.10 Grid search detail

**Figure 2:**
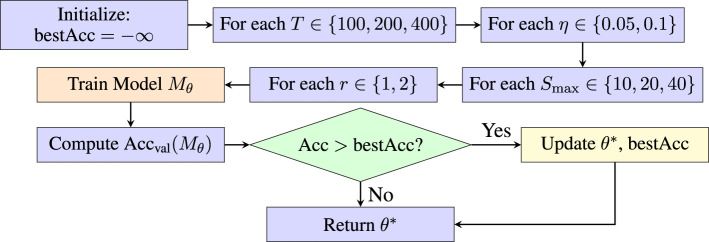
Grid search procedure for hyperparameter optimization.

Figure 2 presents the grid search procedure used in Pipeline B for hyperparameter optimization. The algorithm initializes by setting the best accuracy to negative infinity, then iterates through all combinations of the four hyperparameters: number of learning cycles *T*∈{100, 200, 400}, learning rate η∈{0.05, 0.1}, maximum tree splits *S*_max_∈{10, 20, 40}, and under-sampling ratio *r*∈{1, 2}. For each of the 36 configurations, a RUSBoost model is trained and evaluated on the validation set. If the current configuration achieves higher validation accuracy than the previous best, the optimal configuration θ^*^ and best accuracy are updated. Upon completion, the algorithm returns the hyperparameter configuration that maximized validation performance. The complete pseudocode is provided as Algorithm 3 in the Supplementary material.

### 2.11 Algorithm descriptions

The detailed pseudocode for all algorithms is provided in the Supplementary material.

Algorithm 1: (Learning Curve Generation with Fixed Model Complexity) describes Pipeline A's approach to learning curve construction. This procedure iterates through training fractions from 0.1 to 1.0, training a RUSBoost model with fixed complexity (*T*_sub_ = 120 cycles, η = 0.1, *S*_max_ = 20) at each fraction. For each fraction, stratified sampling creates training subsets, and model performance is averaged over five repetitions to reduce variance from random sampling.

Algorithm 2: (Learning Curve Generation with Scaled Model Complexity) presents Pipeline B's scaled complexity approach. Unlike the fixed-complexity method, this algorithm dynamically adjusts the number of boosting cycles according to $T_{\text{scaled}}=\max\left(30,\left\lfloor T^{*}\cdot f\right\rfloor \right)$, where T^*^ is the optimal number of cycles from grid search and *f* is the training fraction. This scaling ensures model capacity remains proportional to available data, preventing overfitting on small subsets while allowing full model complexity when all training data is used.

Algorithm 3: (Hyperparameter Grid Search) details the systematic search procedure used in Pipeline B. The algorithm exhaustively evaluates all 36 hyperparameter combinations (*T*∈{100, 200, 400}, η∈{0.05, 0.1}, *S*_max_∈{10, 20, 40}, *r*∈{1, 2}) by training a RUSBoost model for each configuration and measuring validation accuracy. The configuration achieving the highest validation accuracy is selected as θ^*^ and used for final model training.

### 2.12 Hyperparameter selection strategy

The fundamental distinction between the two pipelines concerns the approach to hyperparameter determination. Pipeline A assumes that reasonable default values will yield acceptable performance. This assumption proves problematic because the optimal hyperparameter configuration depends on dataset characteristics including size, dimensionality, class balance, and noise level. A configuration that performs well on one dataset may perform poorly on another (40).

Pipeline B addresses this limitation through systematic search. By evaluating 36 candidate configurations on a held-out validation set, the procedure identifies the configuration best suited to the specific dataset. The validation set serves as a proxy for unseen data, reducing the risk of overfitting to idiosyncratic training set properties.

The computational cost of grid search is Equation 13:


$$\begin{array}{c} C_{\text{grid}}=|T|\times |H|\times |S|\times |R|\times C_{\text{train}} \end{array}$$ (13)

where *C*_train_ denotes the cost of training a single model. For the specified search space, this equals 36 × *C*_train_. While this represents a 36-fold increase in training time, the investment yields models with validated hyperparameter choices.

### 2.13 Learning curve construction philosophy

The most consequential difference between the pipelines concerns learning curve generation. This difference directly impacts the interpretability of learning dynamics.

In Pipeline A, every submodel trained during learning curve construction uses *T*_sub_ = 120 boosting cycles regardless of training subset size. Consider the implications of this choice. When training on 10% of the data, the model has access to relatively few examples but employs the same number of boosting iterations as when training on 100% of the data. This mismatch between data availability and model complexity creates artificial conditions that do not reflect realistic learning scenarios.

The consequence is that training accuracy on small subsets may be artificially high because the model has sufficient capacity to memorize the limited training examples. Simultaneously, validation accuracy may appear erratic because the overfitted model generalizes poorly. The resulting learning curves fail to exhibit the smooth, monotonic behavior expected from healthy learning processes.

Pipeline B resolves this issue through proportional scaling. The number of boosting cycles allocated to each submodel grows linearly with the fraction of available data (Equation 14):


$$\begin{array}{c} T_{\text{scaled}}\left(f\right)=\max\left(30,\left\lfloor T^{*}\cdot f\right\rfloor \right) \end{array}$$ (14)

This scaling ensures that model complexity remains commensurate with data availability. With 10% of the data, the model uses approximately 10% of the maximum boosting cycles (subject to the floor of 30 to ensure minimum model capacity). With 50% of the data, the model uses approximately 50% of the cycles. This approach embodies the principle that computational resources should scale with data volume.

The minimum threshold of 30 cycles prevents degenerate models when very small data fractions are used. Without this floor, models trained on minimal data would have insufficient capacity to learn any meaningful patterns.

### 2.14 Under-sampling ratio optimization

Pipeline A uses the default under-sampling ratio of 1, meaning that the majority class is under-sampled to match the minority class exactly during each boosting iteration. Pipeline B includes the ratio as a tunable hyperparameter with candidates *r*∈{1, 2}.

A ratio of 2 allows the majority class to retain twice as many instances as the minority class during under-sampling. This configuration may be beneficial when the majority class contains heterogeneous subgroups that require adequate representation for accurate boundary learning. The optimal ratio depends on the specific class distribution and feature space geometry of the dataset.

### 2.15 Characteristics of healthy learning curves

A properly constructed learning curve should exhibit specific characteristics that indicate sound learning dynamics. The training accuracy curve should start at a high value for small training set sizes, reflecting the model's ability to fit limited data. As training set size increases, training accuracy may decrease slightly as the model encounters more diverse examples that are harder to simultaneously satisfy.

The validation accuracy curve should start lower than training accuracy, reflecting the generalization gap inherent to small training sets. As training set size increases, validation accuracy should increase monotonically, reflecting improved generalization from exposure to more representative training data. The rate of increase typically diminishes as the model approaches its asymptotic performance, producing the characteristic concave shape.

The gap between training and validation curves should narrow as training set size increases. This convergence indicates that the model is learning generalizable patterns rather than memorizing training-set-specific artifacts. Persistent large gaps suggest overfitting, while validation accuracy exceeding training accuracy suggests data leakage or evaluation errors.

### 2.16 Pathological patterns in learning curves

Several patterns indicate unhealthy learning dynamics. Flat training accuracy across all training set sizes suggests that the model has reached its capacity limit regardless of available data. This pattern may indicate insufficient model complexity or features that are not predictive of the target variable.

Decreasing validation accuracy with increasing training set size represents a serious pathology. This pattern suggests that additional training data is degrading rather than improving generalization. Possible causes include concept drift, label noise that accumulates with dataset size, or optimization instabilities.

Non-monotonic validation accuracy, where performance improves then decreases then improves again, indicates unstable learning. Such instability may arise from random variations in under-sampling, high variance in weak learner selection, or hyperparameter settings that create sensitivity to specific training examples.

Extremely large gaps between training and validation accuracy that persist regardless of training set size indicate severe overfitting. This pattern suggests that the model complexity is excessive relative to the information content of the features.

### 2.17 Why scaled complexity produces healthier learning curves

The scaling approach in Pipeline B produces healthier learning curves because it maintains an appropriate balance between model capacity and data availability throughout the learning curve construction process.

Consider the bias-variance tradeoff formalization. The expected prediction error can be decomposed as Equation 15:


$$\begin{array}{c} E\left[{\left(y-\hat{f}\left(\text{x}\right)\right)}^{2}\right]={\text{Bias}}^{2}\left[\hat{f}\left(\text{x}\right)\right]+\text{Var}\left[\hat{f}\left(\text{x}\right)\right]+\sigma^{2} \end{array}$$ (15)

where σ^2^ represents irreducible noise. Model complexity affects both bias and variance. More boosting cycles reduce bias by enabling the ensemble to approximate complex decision boundaries. However, excessive cycles increase variance by fitting noise in the training data.

The optimal number of cycles depends on the amount of training data. With limited data, fewer cycles are needed because the limited examples cannot reliably inform a complex model. With abundant data, more cycles can be productively employed because the model can learn finer distinctions supported by diverse examples.

Pipeline A violates this principle by using fixed complexity regardless of data volume. The result is that small-data models have excessive capacity (high variance, risk of overfitting) while the relationship between model complexity and data availability is obscured in the learning curves.

Pipeline B honors this principle through proportional scaling. Small-data models have appropriately limited capacity, while large-data models have appropriately expanded capacity. The learning curves reflect the true relationship between data availability and model performance, enabling sound diagnostic conclusions.

### 2.18 Evaluation protocol

To compare the two pipelines, both should be executed on the same preprocessed dataset with identical random seed initialization. The comparison should examine the following aspects. Final test set performance metrics including accuracy, precision, recall, and F1-score provide point estimates of model quality. Learning curve shapes reveal the nature of the learning process. Validation accuracy trajectories during training indicate whether the model is improving in the expected manner. Computational cost measurements quantify the overhead of hyperparameter optimization.

### 2.19 Interpretation guidelines

When comparing learning curves from the two pipelines, the following interpretations apply. If Pipeline A produces irregular learning curves while Pipeline B produces smooth, monotonic curves, this indicates that fixed complexity is inappropriate for the dataset. If both pipelines produce similar learning curves, the dataset may be sufficiently simple that hyperparameter choice has limited impact. If Pipeline B produces worse test accuracy despite healthier learning curves, the grid search may have overfit to validation set idiosyncrasies.

The critical principle is that learning curve shape takes precedence over final accuracy in determining result trustworthiness. A model with 90% test accuracy but pathological learning curves should be viewed with more skepticism than a model with 85% test accuracy and textbook learning dynamics (41).

### 2.20 Summary of methodological differences

Table 5 summarizes the principal differences between the two pipelines.

**Table 5:** Comparison of methodological choices between Pipeline A and Pipeline B.

Aspect	Pipeline A (Fixed)	Pipeline B (optimized)
Hyperparameter Selection	Predetermined values	Grid search with validation
Main Model Cycles	150 (fixed)	Selected from {100, 200, 400}
Learning Curve Cycles	120 (fixed for all fractions)	Scaled: max(30, T^*^·*f*)
Learning Rate	0.1 (fixed)	Selected from {0.05, 0.1}
Max Tree Splits	20 (fixed)	Selected from {10, 20, 40}
Under-sampling Ratio	1 (default)	Selected from {1, 2}
Configurations Evaluated	1	36
Learning Curve Rationale	Fixed model complexity	Complexity scales with data

This methodological comparison demonstrates that pipeline design choices significantly impact the interpretability and trustworthiness of machine learning experiments. The two RUSBoost implementations examined differ primarily in their approach to hyperparameter selection and learning curve construction.

Pipeline A employs fixed hyperparameters and constant model complexity during learning curve generation. This approach is computationally efficient but may produce learning curves that do not accurately reflect the relationship between data availability and model performance. The fixed complexity assumption is particularly problematic because it applies excessive model capacity to small training subsets, potentially inflating training accuracy and obscuring true learning dynamics.

Pipeline B incorporates systematic hyperparameter optimization through grid search and scales model complexity proportionally with training data during learning curve construction. The grid search identifies hyperparameters suited to the specific dataset rather than relying on generic defaults. The scaled complexity approach ensures that learning curves reflect realistic conditions where model capacity is matched to data availability.

The analysis emphasizes that learning dynamics must be validated before trusting model results. High accuracy metrics are necessary but not sufficient conditions for model trustworthiness. A model exhibiting pathological learning curves should not be deployed or used for scientific conclusions regardless of its apparent performance on held-out data.

### 2.21 Implementation details

All analyses were conducted using MATLAB R2026a (version 26.1.0.3234472, Update 1) with the Statistics and Machine Learning Toolbox (version 26.1). Complete pseudocode for all preprocessing steps, model training procedures, learning curve construction, and feature importance extraction is provided in the Supplementary material, enabling reproduction of the methodology in any programming environment.

## 3 Results

### 3.1 Baseline algorithm performance metrics

**Figure 3:**
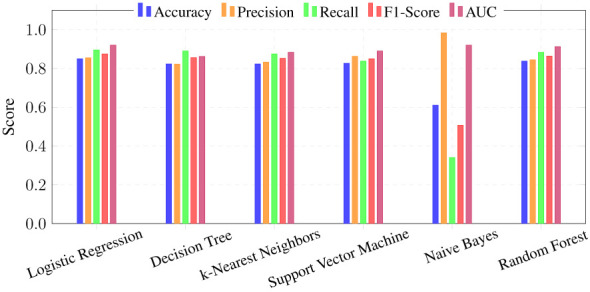
Performance metrics comparison of baseline machine learning algorithms on the student depression dataset. Naive Bayes exhibits anomalous behavior with high precision (0.984) but extremely low recall (0.341), resulting in a poor F1-score (0.507). Logistic Regression achieved the best overall performance with the highest F1-score (0.875) and AUC (0.921).

Figure 3 presents the aggregate performance metrics for the six baseline classification algorithms evaluated on the student depression dataset. Logistic Regression achieved the highest F1-score at 0.875, followed by Random Forest at 0.864, Decision Tree at 0.856, k-Nearest Neighbors at 0.853, and Support Vector Machine at 0.850. Naive Bayes exhibited anomalous behavior with extremely high precision (0.984) but severely degraded recall (0.341), resulting in an F1-score of only 0.507.

Based solely on these aggregate metrics, several algorithms would appear to be acceptable classifiers for the student depression prediction task. Logistic Regression, Random Forest, and Decision Tree all achieved accuracy exceeding 0.82 and F1-scores exceeding 0.85. However, as established in the methodological framework, aggregate metrics provide an incomplete picture of model behavior. A model achieving high test accuracy may nonetheless exhibit pathological learning dynamics that preclude its use for feature importance analysis. The following subsection examines the learning curves for each algorithm to determine whether any demonstrate the healthy learning behavior necessary for trustworthy model interpretation.

### 3.2 Learning curve analysis

**Figure 4:**
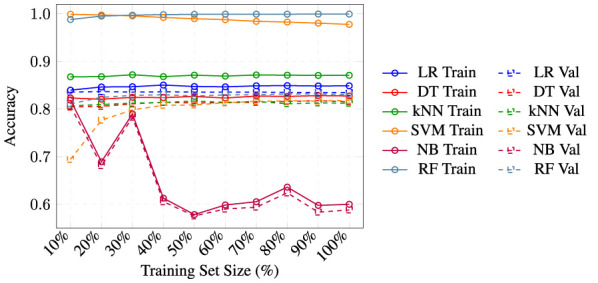
Learning curves for baseline machine learning algorithms. Solid lines represent training accuracy; dashed lines represent validation accuracy. Support Vector Machine and Random Forest exhibit large training-validation gaps indicative of overfitting. Naive Bayes displays erratic, non-monotonic behavior. Logistic Regression demonstrates the healthiest learning dynamics with minimal gap between training and validation curves.

Figure 4 displays the learning curves for all six baseline algorithms, with solid lines representing training accuracy and dashed lines representing validation accuracy across training set sizes ranging from 10% to 100% of the available training data. Examination of these curves reveals that none of the baseline algorithms exhibit healthy learning dynamics.

Logistic Regression and Decision Tree produced essentially flat learning curves for both training and validation accuracy. Training accuracy for Logistic Regression remained constant at approximately 0.84 to 0.85 regardless of training set size, while validation accuracy remained constant at approximately 0.83 to 0.84. Decision Tree exhibited similar behavior with training accuracy stable around 0.82 to 0.83 and validation accuracy around 0.80 to 0.82. Flat learning curves indicate that the algorithms reached their performance ceiling with only 10% of the training data and derived no benefit from the remaining 90%. This pattern suggests either that the features lack sufficient predictive information to support improved performance, or that the algorithms are unable to extract additional signal from larger training sets. In either case, the absence of improvement with increased data volume represents a failure to exhibit the monotonically increasing validation accuracy characteristic of healthy learning.

k-Nearest Neighbors displayed a persistent gap between training and validation accuracy, with training accuracy remaining flat near 0.87 and validation accuracy flat near 0.81. The gap of approximately 0.06 did not diminish as training set size increased, indicating that the algorithm was not converging toward generalizable patterns. The flat trajectories of both curves compound the concern: not only does the gap persist, but neither curve shows the expected response to additional training data.

Support Vector Machine and Random Forest exhibited severe overfitting patterns. Both algorithms achieved near-perfect training accuracy (exceeding 0.97 for SVM and 0.99 for Random Forest) across all training set sizes, while validation accuracy remained substantially lower. The training-validation gap for Support Vector Machine was approximately 0.16 at full training set size, and for Random Forest approximately 0.17. These large gaps that failed to diminish with increased training data indicate that both algorithms memorized training examples rather than learning generalizable patterns. The combination of perfect training accuracy and stagnant validation accuracy is a hallmark of overfitting, rendering these models unsuitable for deployment or scientific interpretation (42).

Naive Bayes displayed the most severe pathological pattern: erratic, non-monotonic learning dynamics. Both training and validation accuracy fluctuated dramatically across training set sizes, with accuracy at 100% training data (approximately 0.60) substantially lower than accuracy at 10% training data (approximately 0.82). A learning curve that decreases with additional training data violates the fundamental principle that more information should improve or at least maintain model performance. This pattern indicates that the distributional assumptions underlying Naive Bayes are fundamentally incompatible with the structure of the student depression dataset.

### 3.3 Summary of baseline algorithm limitations

The learning curve analysis reveals that all six baseline algorithms failed to demonstrate healthy learning dynamics. Table 6 summarizes the pathological patterns observed for each algorithm.

**Table 6:** Summary of pathological learning dynamics observed in baseline algorithms.

Algorithm	Pattern	Interpretation
Logistic Regression	Flat curves	No benefit from additional training data
Decision Tree	Flat curves	No benefit from additional training data
k-Nearest Neighbors	Flat curves with gap	Persistent overfitting, no convergence
Support Vector Machine	Severe overfitting	Memorization rather than generalization
Naive Bayes	Erratic, decreasing	Fundamental model incompatibility
Random Forest	Severe overfitting	Memorization rather than generalization

The flat learning curves observed for Logistic Regression and Decision Tree are particularly concerning because these algorithms achieved the best aggregate metrics among the baseline approaches. High test accuracy combined with flat learning curves suggests that the models are not learning in a manner that can be validated through standard diagnostics. The absence of the expected relationship between training set size and model performance undermines confidence in the models' internal representations, including feature importance rankings.

The observation that no baseline algorithm exhibited healthy learning dynamics motivates the adoption of an alternative approach. Standard classification algorithms, regardless of their aggregate performance, cannot be trusted to produce reliable feature importance rankings when their learning curves fail to demonstrate the expected characteristics of monotonically improving validation accuracy and convergent training-validation gaps (43).

### 3.4 Adoption of RUSBoost

Given the universal failure of baseline algorithms to exhibit healthy learning dynamics, RUSBoost was adopted as an alternative classifier. RUSBoost combines random under-sampling with adaptive boosting, creating balanced training subsets at each iteration while iteratively focusing on difficult-to-classify examples. This approach differs fundamentally from the baseline algorithms in its explicit handling of the training process through iterative reweighting and resampling (44, 45).

The rationale for adopting RUSBoost is not based on an expectation of higher aggregate accuracy, but rather on the hypothesis that its iterative training mechanism may produce more interpretable learning dynamics. If RUSBoost demonstrates the expected relationship between training set size and model performance, with validation accuracy improving monotonically and the training-validation gap converging, then its feature importance rankings can be interpreted with greater confidence than those produced by baseline algorithms with pathological learning curves.

The following sections present the results of applying RUSBoost to the student depression classification task and evaluate whether this alternative approach produces the healthy learning dynamics necessary for trustworthy feature importance analysis.

### 3.5 RUSBoost learning curve comparison

**Figure 5:**
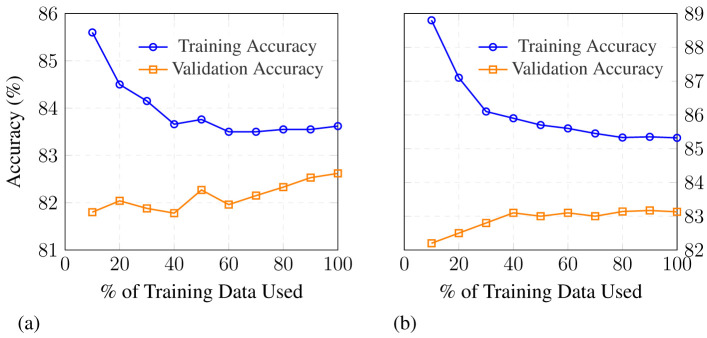
Learning curves for RUSBoost under **(a)** Pipeline A (fixed hyperparameters, left) and **(b)** Pipeline B (tuned hyperparameters, right). Pipeline A exhibits oscillatory validation accuracy that fails to converge smoothly, with irregular fluctuations between 81.75% and 82.65%. Pipeline B demonstrates textbook healthy learning dynamics: training accuracy decreases monotonically from 88.8% to 85.4% as the model encounters more diverse examples, while validation accuracy increases monotonically from 82.2% to 83.1%, with the training-validation gap converging as training set size increases.

Figure 5 presents the learning curves for RUSBoost under both pipeline configurations: Pipeline A with fixed hyperparameters (left panel) and Pipeline B with optimized hyperparameters (right panel). Both implementations demonstrate substantially healthier learning dynamics compared to the baseline algorithms examined in the previous section, yet critical differences between the two configurations reveal the importance of proper hyperparameter tuning and scaled model complexity.

The left panel of Figure 5 displays the learning curves for Pipeline A, which employs fixed hyperparameters (*T*_sub_ = 120, η = 0.1, *S*_max_ = 20) without scaling model complexity to training set size. While RUSBoost under this configuration already outperforms the baseline algorithms by avoiding the severe overfitting observed in Support Vector Machine and Random Forest, the learning dynamics remain suboptimal. The validation accuracy curve exhibits two distinct pathological characteristics. First, it displays oscillatory behavior, fluctuating between approximately 81.75% at 40% training data and 82.65% at full training data rather than increasing monotonically. The validation curve rises from 10% to 20% of training data, decreases at 30% and 40%, rises again at 50%, decreases at 60%, and only then begins a gradual upward trend. Second, and critically, the validation curve never plateaus: at 100% training data, the curve is still rising (82.65%) rather than stabilizing at an asymptotic value. This absence of a plateau indicates that the learning process has not converged, leaving uncertainty about what performance the model would achieve with additional data and suggesting that the model's internal representations may still be evolving. Together, these characteristics (i.e., oscillation and failure to converge) indicate that the fixed-complexity approach produces learning dynamics that cannot be trusted for scientific interpretation. The training-validation gap, while smaller than those observed in baseline algorithms, does not exhibit clear convergence toward zero.

The right panel presents the learning curves for Pipeline B following hyperparameter optimization. The transformation in learning dynamics is striking. After tuning, the RUSBoost model exhibits textbook healthy learning behavior that satisfies all criteria established in the methodological framework. The training accuracy curve begins at 88.8% with 10% of training data and decreases smoothly and monotonically to 85.4% at full training data. This pattern reflects the expected behavior: as the model encounters more diverse training examples, it becomes increasingly difficult to achieve perfect fit on the training set, and training accuracy naturally decreases. Critically, the validation accuracy curve demonstrates the monotonically increasing trajectory characteristic of healthy learning. Starting at 82.2% with minimal training data, validation accuracy rises consistently to 83.1% at full training data, with only minor plateaus rather than the oscillations observed in Pipeline A. The training-validation gap narrows progressively from approximately 6.6 percentage points at 10% training data to approximately 2.3 percentage points at full training data, demonstrating the convergence property essential for trustworthy model interpretation.

The contrast between the two panels illustrates why hyperparameter tuning and scaled model complexity are prerequisites for reliable feature importance analysis. In Pipeline A, the oscillatory validation curve combined with its failure to plateau undermines confidence in the model's learned representations. If the model's generalization performance fluctuates unpredictably with training set size and has not yet converged to a stable asymptote, the feature importance rankings derived from such a model may reflect artifacts of the specific training configuration rather than genuine predictive relationships. In Pipeline B, the smooth, convergent learning curves provide assurance that the model is learning in a principled manner. The monotonically improving validation accuracy indicates that additional training data consistently enhances generalization, while the clear plateau at 83.1% from 70% to 100% training data confirms that the model has reached a stable asymptotic performance. The converging training-validation gap further confirms that the model is extracting generalizable patterns rather than memorizing training-set-specific noise. Table 7 summarizes these distinctions.

**Table 7:** Comparison of learning curve characteristics between Pipeline A (fixed hyperparameters) and Pipeline B (tuned hyperparameters) for RUSBoost.

Characteristic	Pipeline A (fixed)	Pipeline B (tuned)
Validation Accuracy Trajectory	Oscillatory; non-monotonic fluctuations between 81.75% and 82.65%	Monotonically increasing from 82.2% to 83.1%
Validation Curve Convergence	No plateau; still rising at 100% training data (82.65%)	Clear plateau at 83.1% from 70% to 100% training data
Training Accuracy Trajectory	Decreases from 85.6% to 83.65% with minor irregularities	Smooth monotonic decrease from 88.8% to 85.4%
Training-Validation Gap (Initial)	≈3.8 percentage points at 10% data	≈6.6 percentage points at 10% data
Training-Validation Gap (Final)	≈1.0 percentage points at 100% data	≈2.3 percentage points at 100% data
Gap Convergence Behavior	Irregular narrowing with fluctuations	Progressive, monotonic convergence
Learning Dynamics Assessment	Suboptimal; oscillatory and non-convergent	Textbook healthy; expected bias-variance tradeoff behavior with stable plateau
Suitability for Feature Importance Analysis	Unreliable; oscillation and lack of plateau undermine confidence in learned representations	Reliable; smooth convergence and stable plateau support trustworthy interpretation

These results validate the methodological framework established in this study. The scaled complexity approach implemented in Pipeline B, where the number of boosting cycles grows proportionally with training data according to $T_{\text{scaled}}\left(f\right)=\max\left(30,\left\lfloor T^{*}\cdot f\right\rfloor \right)$, ensures that model capacity remains commensurate with data availability throughout the learning curve construction process. This principled scaling, combined with systematic hyperparameter optimization, transforms RUSBoost from an algorithm with merely acceptable learning dynamics into one exhibiting the ideal characteristics necessary for scientific interpretation of feature importance rankings. Consequently, the feature importance analysis presented in subsequent sections is derived from the Pipeline B model, whose healthy learning dynamics establish the foundation for trustworthy conclusions about the predictors of student depression.

### 3.6 RUSBoost aggregate performance metrics

**Figure 6:**
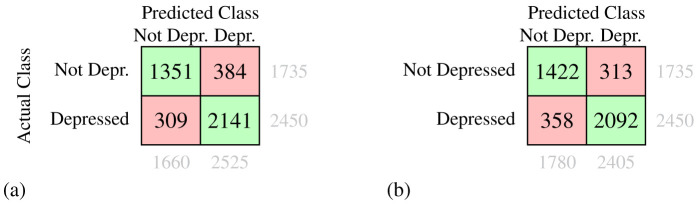
Confusion matrices for RUSBoost under **(a)** Pipeline A (fixed hyperparameters, left) and **(a)** Pipeline B (tuned hyperparameters, right) evaluated on the held-out test set (*n* = 4185). Green cells indicate correct classifications (true negatives and true positives); red cells indicate misclassifications (false positives and false negatives). Gray values along the margins indicate row and column totals. Pipeline B achieves higher true negatives (1422 vs. 1351) and fewer false positives (313 vs. 384), indicating improved specificity, while Pipeline A achieves higher true positives (2141 vs. 2092) and fewer false negatives (309 vs. 358), indicating higher recall.

Having established that Pipeline B produces healthy learning dynamics while Pipeline A exhibits suboptimal oscillatory behavior, this subsection presents the aggregate performance metrics derived from confusion matrices evaluated on the held-out test set. Figure 6 displays the confusion matrices for both pipeline configurations, and Table 8 summarizes the derived performance metrics.

**Table 8:** Aggregate performance metrics derived from confusion matrices for RUSBoost under Pipeline A (fixed hyperparameters) and Pipeline B (tuned hyperparameters).

Metric	Pipeline A (fixed)	Pipeline B (tuned)	Difference
True Positives (TP)	2,141	2,092	−49
True Negatives (TN)	1,351	1,422	+71
False Positives (FP)	384	313	−71
False Negatives (FN)	309	358	+49
Accuracy	83.44%	83.96%	+0.52%
Precision (PPV)	84.79%	86.99%	+2.20%
Recall (Sensitivity)	87.39%	85.39%	−2.00%
Specificity (TNR)	77.87%	81.96%	+4.09%
F1-Score	86.07%	86.18%	+0.11%
Negative Predictive Value (NPV)	81.39%	79.89%	−1.50%
False Positive Rate (FPR)	22.13%	18.04%	−4.09%
False Negative Rate (FNR)	12.61%	14.61%	+2.00%

The confusion matrices reveal a nuanced tradeoff between the two pipeline configurations. Pipeline A with fixed hyperparameters achieved higher recall (87.39% vs. 85.39%), correctly identifying 2,141 of 2,450 depressed students compared to 2092 for Pipeline B. Conversely, Pipeline B achieved substantially higher specificity (81.96% vs. 77.87%), correctly identifying 1,422 of 1,735 non-depressed students compared to 1351 for Pipeline A. This tradeoff manifests in the false positive and false negative counts: Pipeline A produced 384 false positives and 309 false negatives, while Pipeline B produced 313 false positives and 358 false negatives.

The aggregate metrics in Table 8 demonstrate that Pipeline B achieves modestly superior overall performance despite the recall-specificity tradeoff. Accuracy improved from 83.44% to 83.96%, and the F1-score increased marginally from 86.07% to 86.18%. More notably, precision improved substantially from 84.79% to 86.99%, indicating that when Pipeline B predicts a student as depressed, the prediction is more likely to be correct. The 4.09 percentage point improvement in specificity represents a meaningful reduction in false positive classifications, which has practical implications for screening applications where unnecessary interventions carry costs.

The interpretation of these results must be contextualized by the learning curve analysis presented in the preceding subsection. While the aggregate metrics for both pipelines appear comparable, the learning dynamics underlying these results differ fundamentally. Pipeline A's oscillatory validation curve and failure to exhibit monotonic convergence indicate that its aggregate metrics may reflect a fortunate configuration of the specific training-validation-test split rather than robust learned patterns. Pipeline B's textbook healthy learning dynamics (i.e., monotonically decreasing training accuracy, monotonically increasing validation accuracy, and convergent training-validation gap) provide assurance that the model learned in a principled manner.

This distinction has direct implications for feature importance analysis. The feature rankings derived from Pipeline A may be unstable with respect to data perturbations, reflecting the oscillatory learning behavior observed in its learning curves. In contrast, the feature rankings derived from Pipeline B can be interpreted with greater confidence because the smooth, convergent learning dynamics indicate that the model's internal representations are stable and generalizable. Consequently, the feature importance analysis presented in subsequent sections is derived exclusively from the Pipeline B model, whose healthy learning dynamics establish the necessary foundation for trustworthy scientific conclusions about the predictors of student depression.

The slight reduction in recall (from 87.39% to 85.39%) under Pipeline B represents an acceptable tradeoff for the methodological rigor gained. In research applications where the goal is to identify predictive factors rather than to maximize detection of positive cases, the reliability and interpretability of the model take precedence over marginal improvements in sensitivity. The 49 additional false negatives in Pipeline B (358 vs. 309) are counterbalanced by 71 fewer false positives (313 vs. 384), reflecting a more conservative and precise classifier that is better suited for scientific inference.

### 3.7 Sensitivity analysis results

**Figure 7:**
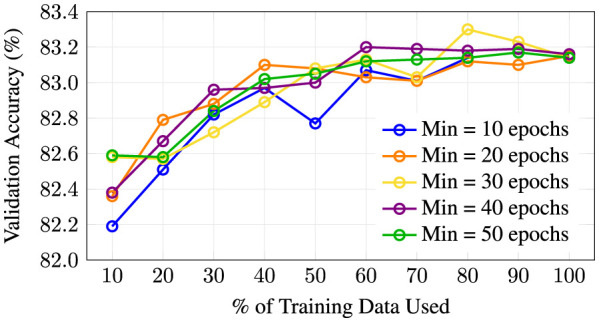
Sensitivity analysis: validation accuracy learning curves across minimum epoch thresholds (10, 20, 30, 40, and 50 epochs). The near-complete overlap of all five curves demonstrates that learning curve characteristics are robust to threshold choice within the tested range. Final validation accuracy varied by only 0.02 percentage points across all thresholds.

Table 9 presents learning curve characteristics across the five tested minimum epoch thresholds, and Figure 7 visualizes the validation accuracy trajectories.

**Table 9:** Sensitivity analysis: learning curve characteristics across minimum epoch thresholds.

Min Threshold	Final Val Acc (%)	Final Gap (%)	Mean Val Acc (%)	Monotonicity Violations
10	83.14	2.18	82.92	3
20	83.15	2.14	82.99	4
30	83.14	2.18	82.98	4
40	83.16	2.16	82.98	2
50	83.14	2.19	82.95	3

Final validation accuracy varied by only 0.02 percentage points across all tested thresholds (range: 83.14% to 83.16%), corresponding to a coefficient of variation of 0.01%. The training-validation gap at full training data ranged from 2.14% to 2.19%, and monotonicity violations ranged from 2 to 4 with no systematic pattern favoring any particular threshold. As illustrated in Figure 7, the validation accuracy trajectories across all five thresholds are nearly superimposed throughout the entire range of training fractions, converging to virtually identical final values. This visual overlap confirms quantitatively that the learning curve characteristics and model performance are robust to the choice of minimum epoch threshold within the tested range. The threshold of 30 epochs was retained as a principled choice that prevents degenerate models on small training fractions while remaining computationally efficient; however, the sensitivity analysis confirms that this specific value does not materially affect the study's conclusions regarding healthy learning dynamics or feature importance rankings.

### 3.8 Feature importance results

**Figure 8:**
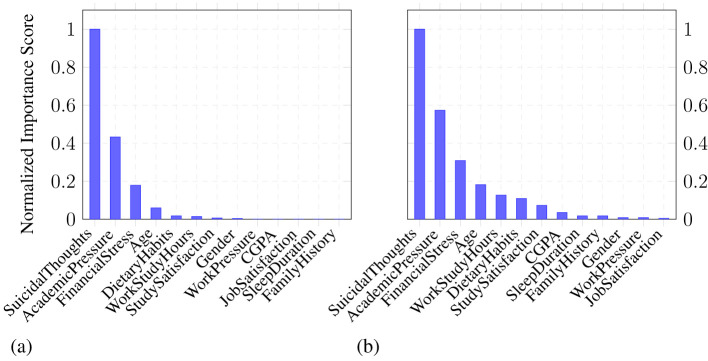
Feature importance rankings for RUSBoost under **(a)** Pipeline A (fixed hyperparameters, left) and **(b)** Pipeline B (tuned hyperparameters, right). Importance scores are normalized to the range [0, 1] by dividing by the maximum importance score in each pipeline. Both configurations identify the same three leading predictors (i.e., history of suicidal thoughts, academic pressure, and financial stress) though the distribution of importance across remaining features differs substantially between pipelines.

Having established that Pipeline B produces healthy learning dynamics while Pipeline A exhibits oscillatory, non-convergent behavior, Figure 8 presents the feature importance rankings derived from both RUSBoost configurations. These rankings quantify the relative contribution of each predictor variable to the classification decision, with higher importance scores indicating greater influence on the model's ability to distinguish between depressed and non-depressed students.

Both pipeline configurations identified the same three features as the dominant predictors of student depression. History of suicidal thoughts (Have You Ever Had Suicidal Thoughts) emerged as the most important predictor in both models, achieving the maximum normalized importance score of 1.0 under both Pipeline A and Pipeline B. Academic pressure ranked second in both configurations, with normalized importance scores of approximately 0.43 (Pipeline A) and 0.57 (Pipeline B). Financial stress ranked third, achieving normalized importance scores of approximately 0.18 (Pipeline A) and 0.31 (Pipeline B).

Beyond the top three predictors, the two pipelines exhibited divergent patterns in how importance was distributed across the remaining features. Under Pipeline B, the tuned model allocated measurable importance to a broader range of variables. Age ranked fourth with a normalized importance score of approximately 0.18, followed by work/study hours (0.13), dietary habits (0.11), study satisfaction (0.07), and CGPA (0.04). Sleep duration, family history of mental illness, gender, work pressure, and job satisfaction all received normalized importance scores below 0.02.

In contrast, Pipeline A concentrated importance more heavily in the top three features, with a steeper decline in importance scores for subsequent predictors. Age received a normalized importance score of approximately 0.06, while dietary habits and work/study hours received scores below 0.02. The remaining features (i.e., study satisfaction, gender, work pressure, CGPA, job satisfaction, sleep duration, and family history of mental illness) all received near-zero normalized importance scores under Pipeline A.

Table 10 summarizes the feature importance rankings for both pipeline configurations, presenting the ordinal rank and approximate importance score for each predictor variable.

**Table 10:** Feature importance rankings comparison between Pipeline A (fixed hyperparameters) and Pipeline B (tuned hyperparameters).

Feature	Pipeline A (fixed)		Pipeline B (tuned)	
	Rank	Normalized Score	Rank	Normalized Score
Suicidal Thoughts History	1	1.000	1	1.000
Academic Pressure	2	0.433	2	0.573
Financial Stress	3	0.179	3	0.309
Age	4	0.060	4	0.182
Work/Study Hours	6	0.015	5	0.127
Dietary Habits	5	0.018	6	0.109
Study Satisfaction	7	0.007	7	0.073
CGPA	10	0.001	8	0.036
Sleep Duration	12	0.001	9	0.018
Family History of Mental Illness	13	0.001	10	0.018
Gender	8	0.004	11	0.009
Work Pressure	9	0.001	12	0.009
Job Satisfaction	11	0.001	13	0.005

Rankings are based on importance scores derived from the trained RUSBoost models. Features are ordered by Pipeline B ranking.

The concentration of importance in Pipeline A, where the top three features account for a larger proportion of total importance compared to Pipeline B, reflects the different learning dynamics observed in the learning curve analysis. Pipeline B's more distributed importance allocation across features is consistent with its stable, convergent learning behavior, which enables the model to extract nuanced predictive signal from multiple variables simultaneously. As established in the methodological framework, only the feature importance rankings from Pipeline B can be considered reliable for scientific interpretation, given that Pipeline A's oscillatory and non-convergent learning dynamics undermine confidence in its learned representations.

## 4 Discussion

### 4.1 The primacy of learning dynamics over aggregate metrics

A comparison of aggregate performance metrics across all evaluated models reveals a counterintuitive finding: the baseline algorithms achieved competitive or superior metrics compared to the RUSBoost implementations. Logistic Regression attained the highest F1-score among all models at 87.5%, exceeding both Pipeline A (86.07%) and Pipeline B (86.18%). Random Forest achieved 86.4%, Decision Tree 85.6%, and k-Nearest Neighbors 85.3%. Even the accuracy metrics tell a similar story: Logistic Regression achieved 85.0% accuracy compared to 83.44% for Pipeline A and 83.96% for Pipeline B. Table 11 summarizes these comparisons.

**Table 11:** Aggregate performance metrics comparison across baseline algorithms and RUSBoost pipelines, with learning dynamics assessment.

Model	Accuracy	F1-Score	Precision	Learning dynamics
Logistic Regression	85.00%	87.50%	85.50%	Flat curves
Random Forest	83.80%	86.40%	84.50%	Severe overfitting
Decision Tree	82.40%	85.60%	82.30%	Flat curves
k-Nearest Neighbors	82.40%	85.30%	83.30%	Persistent gap
Support Vector Machine	82.70%	85.00%	86.30%	Severe overfitting
Naive Bayes	61.10%	50.70%	98.40%	Erratic, decreasing
RUSBoost (Pipeline A)	83.44%	86.07%	84.79%	Oscillatory, non-convergent
RUSBoost (Pipeline B)	83.96%	86.18%	86.99%	Healthy convergence

If model selection were based solely on aggregate metrics, Logistic Regression would be the preferred classifier for the student depression prediction task. Its F1-score exceeds that of both RUSBoost configurations, and its accuracy surpasses Pipeline B by more than one percentage point. By conventional evaluation standards, the computationally intensive hyperparameter optimization and scaled complexity approach implemented in Pipeline B would appear to have been wasted effort, yielding a model that underperforms a simple linear classifier on standard benchmarks.

However, this interpretation fundamentally misunderstands the purpose of machine learning validation. Aggregate metrics summarize model performance at a single point in time on a single held-out dataset. They reveal nothing about the process by which that performance was achieved, the stability of the learned representations, or the reliability of the model's internal structure for scientific interpretation. A model may achieve high accuracy through memorization of training examples, through exploitation of spurious correlations specific to the particular train-test split, or through genuine learning of generalizable patterns. Aggregate metrics cannot distinguish among these possibilities.

Learning curve analysis provides this distinction (36). The flat curves exhibited by Logistic Regression indicate that the algorithm derived no benefit from 90% of the available training data, a pattern inconsistent with genuine learning from the data's predictive structure. The severe overfitting observed in Random Forest and Support Vector Machine, with training accuracy exceeding 97% while validation accuracy stagnated below 83%, indicates memorization rather than generalization. The erratic trajectories of Naive Bayes, with performance declining as training data increased, reveal fundamental incompatibility between model assumptions and data structure. Even Pipeline A's RUSBoost implementation, despite achieving reasonable aggregate metrics, exhibited two distinct pathological characteristics: oscillatory validation accuracy that fluctuated unpredictably across training fractions, and failure to plateau, with the validation curve still rising at 100% training data rather than stabilizing at an asymptotic value. Together, these patterns indicate that Pipeline A's learning process was both unstable and non-convergent, undermining confidence in the reliability of its learned representations.

Only Pipeline B demonstrated the characteristics of healthy learning: monotonically decreasing training accuracy as the model encountered increasingly diverse examples, monotonically increasing validation accuracy as additional data improved generalization, and progressive convergence of the training-validation gap indicating that the model was extracting stable, generalizable patterns rather than fitting to noise. These dynamics provide assurance that the model's internal structure (including its feature importance rankings) reflects genuine predictive relationships rather than artifacts of the specific training configuration.

### 4.2 Establishing the foundation for feature importance analysis

Feature importance rankings derived from models with pathological learning dynamics cannot be trusted, regardless of how favorable their aggregate metrics appear. As Section 4.1 demonstrated, the baseline algorithms' learning curves indicate that their internal representations are unreliable, i.e., coefficients or importance scores from these models may reflect patterns learned from small data subsets, memorization artifacts, or spurious correlations rather than generalizable predictive signal. This reasoning reinforces the principle established above, that learning dynamics must take precedence over aggregate metrics in determining model trustworthiness.

Consequently, despite achieving neither the highest accuracy nor the highest F1-score among the evaluated models, Pipeline B's RUSBoost implementation is the only model whose learning dynamics support confident interpretation of its internal structure. The baseline algorithms, regardless of their superior aggregate metrics, cannot be trusted for scientific inference because their pathological learning dynamics preclude meaningful assessment of what patterns they actually learned.

### 4.3 Feature importance as a clinical validation tool

Beyond its primary role in identifying predictive factors, feature importance analysis serves as a critical validation tool for assessing the clinical plausibility of a trained machine learning model. A classifier that has genuinely learned from the data should produce feature importance rankings that align with established clinical and epidemiological knowledge. At minimum, the features identified as predictive should make sense from a domain perspective: their order and relative magnitudes should reflect plausible relationships between predictors and outcome. When a model assigns importance scores that contradict well-established clinical relationships, this inconsistency signals that the model's learned representations may be unreliable, regardless of its aggregate performance metrics.

Examining the feature importance results through this clinical validation lens reveals important distinctions between the two pipeline configurations. Under Pipeline B, the tuned model allocated measurable importance to a range of clinically relevant variables. History of suicidal thoughts emerged as the dominant predictor, followed by academic pressure, financial stress, age, work/study hours, dietary habits, study satisfaction, and CGPA. This distribution is clinically coherent: suicidal ideation is strongly comorbid with depression, academic and financial stressors are well-documented risk factors in student populations, and lifestyle factors such as diet and sleep have established associations with mental health outcomes. Sleep duration, family history of mental illness, and demographic variables received smaller but non-negligible importance scores, consistent with their established but secondary roles in depression etiology.

In contrast, Pipeline A's importance distribution raises concerns about clinical plausibility. While the top three predictors (suicidal thoughts history, academic pressure, and financial stress) align with Pipeline B and clinical expectations, the remaining features received near-zero importance scores. Variables such as sleep duration, family history of mental illness, CGPA, and study satisfaction (all of which have documented associations with depression in the literature) were effectively assigned negligible predictive value by Pipeline A. This pattern is difficult to reconcile with clinical knowledge. Sleep disturbance is both a core symptom and established predictor of depression. Family history of mental illness is a recognized risk factor with substantial genetic and environmental components. Academic performance and satisfaction are known correlates of student mental health. A model that assigns essentially zero importance to these clinically relevant factors fails a basic test of face validity, suggesting that its learned representations do not accurately capture the predictive structure of the data.

This discrepancy between Pipeline A and Pipeline B provides additional validation for the conclusions drawn from learning curve analysis. Pipeline A's oscillatory, non-convergent learning dynamics produced a model that concentrated importance excessively in a small subset of features while failing to extract predictive signal from other clinically relevant variables. Pipeline B's healthy, convergent learning dynamics enabled the model to learn a more nuanced representation that appropriately weights multiple contributing factors in a manner consistent with clinical understanding.

As established in Section 4.2, the baseline algorithms' pathological learning dynamics preclude meaningful feature importance analysis; interpreting their feature rankings would constitute an exercise in analyzing artifacts rather than discovering scientific insights. The RUSBoost implementations, while imperfect under Pipeline A, at least produced feature rankings that could be examined for clinical plausibility. Pipeline A's near-zero importance scores for clinically relevant variables served as a diagnostic signal that the model's learning process was suboptimal, even though its aggregate metrics appeared acceptable. This observation reinforces the utility of feature importance analysis as a validation tool: clinical implausibility in feature rankings can reveal problems with model training that aggregate metrics alone would obscure.

The feature importance rankings from Pipeline B therefore represent the only scientifically defensible basis for drawing conclusions about the predictors of student depression in this dataset, given that it is the only model where healthy learning dynamics and clinically coherent feature rankings converge.

### 4.4 Implications for medical student mental health interventions

The feature importance rankings derived from Pipeline B provide empirically grounded guidance for institutional priorities in supporting medical student mental health. The identification of suicidal ideation history as the dominant predictor (normalized importance: 1.0) underscores the critical importance of routine, confidential screening for suicidal thoughts during medical training. Students experiencing suicidal ideation may not voluntarily disclose these thoughts due to concerns about stigma, fears of being removed from training, or beliefs that such thoughts represent personal failure. Systematic screening protocols that normalize mental health assessment and clearly separate clinical support from academic evaluation can create pathways for early identification and intervention.

The prominence of academic pressure (normalized importance: 0.57) as the second-ranked predictor aligns with the qualitative understanding of medical education stressors. The transition from undergraduate to medical education involves not merely an increase in workload volume but a fundamental shift in the nature and pace of learning. Students who excelled in environments where material was presented sequentially and mastered through repetition often struggle when faced with interleaved topics, case-based reasoning, and the expectation of integrating knowledge across domains in real time. Institutions might consider workload management strategies including curriculum pacing reforms, explicit instruction in metacognitive study strategies appropriate for medical education, and structured peer learning programs that reduce the isolation students feel when struggling with difficult material.

Financial stress (normalized importance: 0.31) represents a structural barrier that institutions cannot fully resolve but can partially mitigate. Beyond traditional financial aid mechanisms, institutions can provide emergency financial assistance funds, facilitate connections to financial planning resources, and structure curricula to minimize additional costs such as required travel for clinical rotations or expensive examination preparation materials. Transparency about the total cost of attendance and realistic projections of post-graduation debt burden can help students make informed decisions early in their training, reducing the sense of financial entrapment that develops when students realize mid-program that their debt load will be far higher than anticipated.

The ranking of age (normalized importance: 0.18), work/study hours (0.13), dietary habits (0.11), and study satisfaction (0.07) as secondary predictors suggests that lifestyle factors and student circumstances contribute meaningfully to depression risk, though not as dominantly as suicidal ideation history and academic pressure. This finding supports holistic wellness initiatives that address sleep, nutrition, and work-life balance alongside academic support. The relatively lower importance of family history of mental illness (0.018) and demographic factors such as gender (0.009) indicates that while these variables may have clinical relevance in individual cases, they do not emerge as strong population-level predictors in this dataset, suggesting that the proximal stressors of medical education environment dominate the distal risk factors typically emphasized in psychiatric epidemiology.

These findings must be interpreted in light of significant barriers that at-risk medical students face in accessing mental health support. High-achieving individuals who have built their identities around academic success may view mental health struggles as personal failure rather than as expectable responses to extreme environmental stress (46, 47). However, even when students recognize the need for support, the very academic pressures identified as the second-strongest predictor in this study create a practical barrier: many students express interest in obtaining mental health services but feel they lack time for therapy, particularly the long-term therapeutic relationships where treatment is typically most effective (21, 48).

From a screening perspective, many mental health surveys are lengthy, often exceeding 10 minutes and combining multiple-choice and short-answer questions. These surveys may fail to prioritize the most predictive factors (49, 50). The feature importance rankings from this study highlight an opportunity to develop more efficient screening protocols. Streamlined check-in questionnaires, focused specifically on the variables identified as most predictive (such as suicidal ideation history, academic pressure, financial stress, and key lifestyle factors) could provide clinically meaningful assessments in a fraction of the time. Such abbreviated instruments would reduce the burden of participation while maintaining, or potentially improving, screening sensitivity by concentrating on high-signal predictors.

The effectiveness of streamlined screening depends critically on coupling rapid assessment with prompt connection to appropriately matched support services. Students need access not merely to generic mental health resources, but specifically to clinicians and support systems that understand the unique pressures of medical education: the cognitive load of integrating vast knowledge domains, the emotional weight of clinical responsibilities, the identity challenges of transitioning from top undergraduate performers to struggling trainees, and the financial constraints that limit their options for self-care (51). Creating institutional cultures that explicitly normalize mental health challenges, provide confidential access to such specialized support services, and separate clinical mental health care from academic performance evaluation can reduce both psychological and practical barriers (21, 48, 52). The machine learning approach demonstrated in this study offers a foundation for these streamlined screening mechanisms that do not require students to self-identify, though any implementation would need to carefully address privacy concerns, ensure that screening is coupled with adequate support resources, and avoid creating additional sources of anxiety through surveillance.

From an implementation perspective, the intended users of such a predictive model would be institutional wellness coordinators, student affairs professionals, and mental health service administrators rather than clinicians making individual diagnostic decisions. The model would function as a population-level triage tool to identify students who might benefit from outreach, not as a clinical diagnostic instrument. Screening could occur at defined timepoints such as matriculation, the beginning of each academic year, and following high-stress periods such as examinations. False positives, wherein students flagged by the model do not have clinical depression, would result in students receiving outreach for support services they may not need; the cost of such errors is low provided outreach is framed as routine wellness contact rather than as identification of pathology. False negatives, wherein students with depression are not flagged, represent a more serious concern; accordingly, model-based screening should supplement rather than replace existing self-referral pathways and routine clinical screening. Privacy concerns are substantial because students may fear that mental health data could affect academic standing or future career opportunities; any implementation must ensure strict separation between predictive screening data and academic records, with explicit institutional policies prohibiting adverse academic or professional consequences from mental health disclosures.

### 4.5 Limitations

The Student Depression Dataset comprises a general student population rather than medical students specifically; accordingly, the feature importance findings are applicable to students across diverse academic disciplines, as the manuscript title indicates. The Discussion emphasizes implications for medical students because the authors, writing from a medical education context, are most familiar with the unique challenges and institutional structures of medical training. This represents contextualized interpretation for an audience the authors serve directly, not a restriction of the findings to medical trainees. Researchers and administrators in other academic settings may apply these results to their respective student populations.

The current study demonstrates the importance of validated learning dynamics for trustworthy feature importance analysis in mental health prediction. However, several limitations suggest directions for future research. First, the Student Depression Dataset, while comprehensive in its coverage of demographic, academic, and psychological factors, lacks explicit measures of physical health symptoms. This represents a significant gap given that depression and stress often manifest through psychosomatic pathways. Students experiencing mental health challenges may present with physical complaints (including chronic fatigue, headaches, gastrointestinal issues, musculoskeletal pain, or sleep disturbances) before recognizing or reporting psychological symptoms (53, 54). Current screening approaches that rely exclusively on psychological self-report measures may miss individuals whose depression primarily expresses itself through somatic channels (55–57).

The present analysis did not compare model performance against established screening instruments such as the Patient Health Questionnaire (PHQ-9) or against clinician judgment; however, such comparison was not the objective of this study. The PHQ-9 is a validated diagnostic and severity-monitoring tool that asks patients directly about depressive symptoms over a defined recall period (58–60). In contrast, the present study sought to train a machine learning classifier on routinely collected student data, validate that the model exhibited healthy learning dynamics, and subsequently extract feature importance rankings to identify which variables most strongly predict depression status across the dataset as a whole. This objective differs fundamentally from the diagnostic purpose served by instruments like the PHQ-9. Traditional statistical approaches such as correlation analysis or univariate regression examine relationships between individual predictors and the outcome in isolation, quantifying pairwise associations without accounting for how predictors jointly and interactively contribute to classification. Machine learning, by contrast, constructs a multivariate decision function that leverages all features simultaneously; the resulting feature importance scores reflect each variable's contribution within this joint predictive context, capturing interactions and redundancies that individual statistical tests cannot detect. Consequently, the clinical value of this analysis lies not in replacing validated screening tools but in providing an empirically grounded ranking of modifiable and non-modifiable factors associated with student depression, thereby informing institutional priorities for prevention and early intervention. Any future deployment of the model as a screening instrument would require prospective head-to-head evaluation against the PHQ-9 and clinician judgment to establish comparative diagnostic accuracy, but such deployment was beyond the scope of the present methodological investigation.

The study relies on a single public dataset with a random train/validation/test split and lacks external validation on independent cohorts, different time periods, or alternative questionnaire implementations. External validation is critical for assessing generalizability and is explicitly encouraged by the TRIPOD+AI reporting guidelines (61, 62). However, the absence of external validation reflects a common constraint in machine learning research: truly external datasets (i.e., those collected independently with different instruments, populations, or time periods) are frequently unavailable, and randomly splitting a single dataset does not constitute external validation regardless of labeling conventions. This limitation is compounded by the synthetic nature of the Student Depression Dataset: because the data were algorithmically generated rather than collected from any real-world population, the dataset has no geographic provenance, and findings cannot be assumed to generalize to authentic student populations in any specific region or institutional context.

In the absence of external data, it is standard machine learning practice to assess potential generalizability through learning curve analysis, as presented in this study. Learning curves that demonstrate monotonically increasing validation accuracy, convergent training-validation gaps, and stable asymptotic performance provide evidence that the model is extracting generalizable patterns rather than memorizing dataset-specific artifacts (38, 39). While learning curve diagnostics do not guarantee unbiased performance on truly novel populations, they represent the next best evidence for generalizability when external validation is infeasible. It is important to distinguish between data partitioning strategies when evaluating internal validation approaches. K-fold cross-validation is particularly valuable when a 2-way split (train/test) is employed, as it preserves data while providing robust performance estimates through averaging across folds. TRIPOD+AI guidelines recommend internal resampling techniques (e.g., cross-validation) as a prerequisite for prediction model development, particularly when data are limited. The present study employed a 3-way split (train/validation/test) with a dataset of 27,901 records, yielding approximately 19,500 training samples, 4,200 validation samples, and 4,200 test samples. With data of this magnitude, the dedicated validation set provides stable estimates for hyperparameter selection without requiring the data-preserving benefits of cross-validation. The 3-way split design ensures that hyperparameter tuning (validation set) remains strictly independent of final performance evaluation (test set), preventing information leakage between model selection and assessment. Nevertheless, generalizability remains uncertain until the methodology is validated on independent real-world datasets, which represents the definitive standard for external validity.

Model calibration (i.e., the correspondence between predicted probabilities and observed outcome frequencies) was not evaluated because the study objective was feature importance ranking rather than probability-based individualized risk prediction. Hence, the present study does not meet all TRIPOD+AI criteria for clinical prediction model reporting (e.g., external validation on independent cohorts, calibration assessment); however, the study is framed as a methodological demonstration of learning curve diagnostics for trustworthy feature importance analysis rather than a clinical prediction tool ready for deployment.

Notably, a substantial proportion of published machine learning studies in health-related domains report high aggregate performance metrics without presenting learning curves or other diagnostics of training dynamics. In such cases, favorable final metrics may arise from overfitting, data leakage, or other artifactual issues rather than from principled learning, yet these concerns remain undetectable without explicit validation of the training process. By presenting comprehensive learning curve analyses demonstrating healthy training dynamics, the present study provides stronger evidence of potential generalizability than studies reporting only final metrics. Validation on authentic clinical datasets from diverse populations remains essential before any practical application of these findings.

The present analysis did not explicitly model interaction terms or nonlinear correlations among predictor variables. While ensemble methods such as RUSBoost can implicitly capture some nonlinear relationships through their tree-based weak learners, the study did not systematically investigate whether specific feature interactions (e.g., academic pressure combined with sleep deprivation, or financial stress interacting with dietary habits) provide additional predictive signal beyond main effects.

Other additional limitations warrant acknowledgment. The dataset does not capture comorbid conditions such as anxiety disorders, substance use, or prior mental health treatment, all of which may confound or moderate the relationship between predictors and depression (63, 64). Both predictors and the depression outcome are self-reported without clinical verification, introducing potential reporting bias (65, 66).

### 4.6 Future directions

Future work should expand depression prediction datasets to include comprehensive physical symptom inventories alongside traditional mental health indicators. Machine learning models trained on such multimodal datasets could identify latent associations between specific patterns of physical symptoms and underlying depression or stress. For example, the combination of chronic headaches, sleep disruption, and digestive complaints might emerge as a reliable predictor of undiagnosed depression, even in the absence of explicitly reported psychological distress. Such models would enable more holistic screening protocols that recognize the interconnected nature of physical and mental health in student populations.

The pattern-seeking capabilities of modern machine learning algorithms make them particularly well-suited for uncovering these psychosomatic relationships. Traditional statistical approaches typically require researchers to specify hypothesized associations a priori. In contrast, algorithms such as gradient boosting machines and neural networks can discover complex, nonlinear relationships between physical symptom profiles and mental health outcomes without requiring explicit specification of which physical symptoms might be relevant. This data-driven discovery process could reveal previously unrecognized somatic presentations of depression that vary across demographic subgroups or cultural contexts.

External validation on independent cohorts remains the gold standard for establishing model generalizability and should be pursued when appropriate datasets become available. Such validation would ideally involve student populations from different institutions, geographic regions, time periods, or questionnaire implementations to assess whether the feature importance rankings and predictive relationships identified in this study replicate across diverse contexts.

Subgroup-specific modeling represents an important avenue for future research. The present study trained a single model on the entire dataset without stratification by demographic variables such as gender or age group. While subgroup analyses are standard in traditional statistical investigations, machine learning models require substantially larger sample sizes to achieve stable learning dynamics. Partitioning the dataset by demographic subgroups would proportionally reduce the training data available for each subgroup-specific model, likely resulting in insufficient samples to demonstrate healthy learning curves. Under such conditions, subgroup models would exhibit the same pathological dynamics (flat curves, erratic validation accuracy, persistent overfitting) observed in the baseline algorithm evaluation, rendering their feature importance rankings untrustworthy. When larger datasets become available, researchers should train and validate separate models for demographic subgroups, explicitly reporting learning curve diagnostics for each to confirm that subgroup-specific findings rest on demonstrably healthy training processes. Until such data are available, the feature importance rankings reported in this study should be interpreted as population-level findings that may not uniformly apply across all demographic strata.

Explicit modeling of interaction terms and nonlinear correlations among predictor variables represents another avenue for future research. Feature engineering approaches including polynomial features and multiplicative interaction terms, as well as algorithms specifically designed to detect interaction effects, could potentially improve predictive performance and yield more nuanced insights into the combinatorial pathways through which multiple stressors jointly influence depression risk. Future work should explore whether latent correlational structures among predictors enhance model performance and interpretability through polynomial features, interaction terms, and nonlinear dimensionality reduction techniques.

Additionally, systematic ablation experiments would help quantify the independent and synergistic contributions of the two core design elements of Pipeline B: hyperparameter grid search and model complexity scaling with data availability. A full factorial design comparing pipeline variants with neither, one, or both components would clarify which element is primarily responsible for producing healthy learning dynamics and guide practitioners in selecting appropriate configurations for their specific applications.

## 5 Conclusion

This study demonstrates that rigorous validation of learning dynamics is an essential prerequisite for trustworthy machine learning-based analysis of student depression predictors, and that aggregate performance metrics alone are insufficient indicators of model reliability. While baseline algorithms including Logistic Regression achieved higher F1-scores than the RUSBoost implementations, their pathological learning curves (characterized by flat trajectories, severe overfitting, or erratic non-monotonic behavior) precluded meaningful interpretation of their internal representations. Only the optimized RUSBoost pipeline (Pipeline B), which incorporated systematic hyperparameter tuning and scaled model complexity proportional to training data availability, exhibited the textbook healthy learning dynamics necessary for scientific inference, i.e., monotonically increasing validation accuracy, convergent training-validation gaps, and stable asymptotic performance. For medical students specifically, these findings underscore the importance of early identification systems that do not rely solely on self-disclosure, given that high-achieving students often face psychological barriers to seeking help. The bidirectional relationship between academic difficulties and mental health deterioration creates a self-reinforcing cycle that interventions must interrupt early through proactive screening, normalized access to support services, and institutional cultures that reframe mental health challenges as expectable responses to demanding training environments rather than as indicators of personal inadequacy.

Feature importance analysis derived from this validated model identified history of suicidal thoughts, academic pressure, and financial stress as the dominant predictors of student depression, with secondary contributions from age, work/study hours, dietary habits, and study satisfaction; which is a clinically coherent ranking that aligns with established epidemiological understanding. These findings have direct implications for educational institutions and mental health practitioners: targeted interventions addressing academic workload management and financial support programs may prove most effective in reducing depression prevalence among student populations. More broadly, this work establishes a methodological framework emphasizing that learning curve diagnostics must precede and inform any interpretation of machine learning model outputs, ensuring that scientific conclusions about predictive factors rest on demonstrably reliable learned representations rather than artifacts of pathological training processes.

## Data Availability

The original contributions presented in the study are included in the article/Supplementary material, further inquiries can be directed to the corresponding author.
