# Supplementary material for: Identifying predictors of student depression through validated machine learning pipelines
Source: Front Med (Lausanne). 2026 Jun 29;13:1864665. doi: 10.3389/fmed.2026.1864665 (PMC13357854; doi:10.3389/fmed.2026.1864665)
Supplement: Supplementary file 1 [file Data_Sheet_1.pdf]

Supplementary Material

1 PIPELINE A: FIXED CONFIGURATION LEARNING CURVE GENERATION

Algorithm 1 Learning Curve Generation with Fixed Model Complexity

Require: Training data $(X_{\text{train}}, Y_{\text{train}})$, Validation data $(X_{\text{val}}, Y_{\text{val}})$
Require: Fixed parameters: $T_{\text{sub}} = 120$, $\eta = 0.1$, $S_{\max} = 20$
Ensure: Training and validation accuracy vectors

fractions $\leftarrow [0.1, 0.2, \ldots, 1.0]$
trainAcc $\leftarrow$ zeros$(|$**fractions**$|)$
valAcc $\leftarrow$ zeros$(|$**fractions**$|)$
numRepeats $\leftarrow 5$
for $i = 1$ to $|$**fractions**$|$ **do**
    $f \leftarrow$ **fractions**$[i]$
    $n \leftarrow \max(10, \lfloor f \cdot |Y_{\text{train}}| \rfloor)$
    tempTrain $\leftarrow$ zeros(numRepeats)
    tempVal $\leftarrow$ zeros(numRepeats)
    for $r = 1$ to numRepeats **do**
        $(X_{\text{sub}}, Y_{\text{sub}}) \leftarrow$ StratifiedSample$(X_{\text{train}}, Y_{\text{train}}, n)$
        $w_{\text{sub}} \leftarrow$ ComputeClassWeights(Y_{sub})
        $M \leftarrow$ TrainRUSBoost$(X_{\text{sub}}, Y_{\text{sub}}, T_{\text{sub}}, \eta, S_{\max}, w_{\text{sub}})$
        tempTrain$[r] \leftarrow$ Accuracy$(M, X_{\text{sub}}, Y_{\text{sub}})$
        tempVal$[r] \leftarrow$ Accuracy$(M, X_{\text{val}}, Y_{\text{val}})$
    end for
    trainAcc$[i] \leftarrow$ mean(tempTrain)
    valAcc$[i] \leftarrow$ mean(tempVal)
end for
return trainAcc, **valAcc**

2 PIPELINE B: SCALED COMPLEXITY LEARNING CURVE GENERATION

Algorithm 2 Learning Curve Generation with Scaled Model Complexity

Require: Training data $(X_{\text{train}}, Y_{\text{train}})$, Validation data $(X_{\text{val}}, Y_{\text{val}})$
Require: Optimized parameters: T^*, η^*, $S^*_{\max}$, r^*
Ensure: Training and validation accuracy vectors

fractions $\leftarrow [0.1, 0.2, \ldots, 1.0]$
trainAcc $\leftarrow$ zeros(|**fractions**|)
valAcc $\leftarrow$ zeros(|**fractions**|)
numRepeats $\leftarrow 5$
for $i = 1$ to |**fractions**| **do**
    $f \leftarrow$ **fractions**$[i]$
    $n \leftarrow \max(20, \lfloor f \cdot |Y_{\text{train}}| \rfloor)$
    $\mathbf{T}_{\text{scaled}} \leftarrow \max(\mathbf{30}, \lfloor \mathbf{T}^* \cdot \mathbf{f} \rfloor)$ ▷ Key difference
    tempTrain $\leftarrow$ zeros(numRepeats)
    tempVal $\leftarrow$ zeros(numRepeats)
    for $r = 1$ to numRepeats **do**
        $(X_{\text{sub}}, Y_{\text{sub}}) \leftarrow$ StratifiedSample$(X_{\text{train}}, Y_{\text{train}}, n)$
        $w_{\text{sub}} \leftarrow$ ComputeClassWeights(Y_{sub})
        $M \leftarrow$ TrainRUSBoost$(X_{\text{sub}}, Y_{\text{sub}}, \mathbf{T}_{\text{scaled}}, \eta^*, S^*_{\max}, w_{\text{sub}}, r^*)$
        tempTrain$[r] \leftarrow$ Accuracy$(M, X_{\text{sub}}, Y_{\text{sub}})$
        tempVal$[r] \leftarrow$ Accuracy$(M, X_{\text{val}}, Y_{\text{val}})$
    end for
    trainAcc$[i] \leftarrow$ mean(tempTrain)
    valAcc$[i] \leftarrow$ mean(tempVal)
end for
return trainAcc, **valAcc**

3 GRID SEARCH PROCEDURE

Algorithm 3 Hyperparameter Grid Search

Require: Training data $(X_{\text{train}}, Y_{\text{train}})$, Validation data $(X_{\text{val}}, Y_{\text{val}})$
Require: Search space: $\mathcal{T}, \mathcal{H}, \mathcal{S}, \mathcal{R}$
Ensure: Optimal hyperparameter configuration θ^*

bestAcc $\leftarrow -\infty$
$\theta^* \leftarrow$ null
$w \leftarrow$ ComputeClassWeights(Y_{train})
for $T \in \mathcal{T}$ **do**
    for $\eta \in \mathcal{H}$ **do**
        for $S_{\max} \in \mathcal{S}$ **do**
            for $r \in \mathcal{R}$ **do**
                $M \leftarrow$ TrainRUSBoost$(X_{\text{train}}, Y_{\text{train}}, T, \eta, S_{\max}, w, r)$
                $\hat{Y}_{\text{val}} \leftarrow$ Predict(M, X_{val})
                acc $\leftarrow \frac{1}{|Y_{\text{val}}|} \sum_i \mathbf{1}[\hat{Y}_{\text{val},i} = Y_{\text{val},i}]$
                if acc $>$ bestAcc **then**
                    bestAcc $\leftarrow$ acc
                    $\theta^* \leftarrow (T, \eta, S_{\max}, r)$
                end if
            end for
        end for
    end for
end for
return θ^*
